# Communicative Functions in Children Raised in Three Different Social Contexts in Colombia: The Key Issue of Joint Attention

**DOI:** 10.3389/fpsyg.2021.642242

**Published:** 2021-07-15

**Authors:** Mayilín Moreno, Evelyne Thommen, Elianne Morán, Michèle Guidetti

**Affiliations:** ^1^Research Group in Psychology, Cognition, Communication and Development (CCD), Universidad del Norte, Barranquilla, Colombia; ^2^Haute École de Travail Social et de la Santé (HETSL), University of Applied Sciences and Arts Western Switzerland, Lausanne, Switzerland; ^3^Cognition, Langues, Langage, Ergonomie (CLLE), Université de Toulouse, CNRS, UT2J, Toulouse, France

**Keywords:** social interaction, joint attention, behavior regulation, socioeconomic contexts, Colombia

## Abstract

Children’s sociocultural experiences vary around the world. Colombia is a South American country where the differences between socioeconomic statuses (SES) are huge. In this study, through the ECSP-E Scale, translated to Spanish and validated for linguistic and cultural equivalence, the development of three communicative functions was evaluated through an interactive sociopragmatic approach. The participants comprised 36 24-month-old children, raised in three different social contexts in Colombia, with the goal of comparing them across groups of SES. The lowest SES group sample subjects were representative of extreme poverty and members of an ethnic group, the Wayuú. Results for the communicative functions, namely social interaction (SI), joint attention (JA), and behavior regulation (BR), showed that the only function with no significant differences across SES was joint attention. This supports the hypothesis that the development of this function may be universal, in light of the fact that the Wayuú not only differed from other subjects in terms of their socioeconomic status but also in their culture. Higher SES was related to better social interaction, while Low SES was associated with better behavior regulation than their High SES peers. Consequently, results are discussed considering socioeconomic and cultural differences in the development of communication and social interactions, leading us to reexamine the paradigms, theories, and practices that are used when observing children raised in very poor environments.

## Introduction

The child’s first communications with his environment are evidently non-verbal and occur in the middle of interactive sequences with the adult (generally the mother). This interactive duo is essential for both language development and the child’s socio-cognitive development (Bruner, 1983; Song et al., 2014; Tamis-LeMonda et al., 2014; Donelly and Kidd, 2021). Indeed, in terms of early language development, some studies suggest that the response capacity of parents to the communicative behaviors of babies predicts the learning of words (Tamis-LeMonda et al., 2014). Likewise, the interactive relationship between the child and the adult facilitates learning of the first concepts, that provide the child with the referents on which he can map the first words, thus organizing and consolidating his knowledge (Song et al., 2014). In this sense, adults provide a scaffolding or support that facilitates and promotes children’s learning and development.

The sociopragmatic approach of development, which includes the works of Bates et al. (1979), Bruner (1983), and those related to usage based theory of language acquisition (Tomasello, 2003) sustains that there is a continuity between prelinguistic and linguistic development in children: the expressive behaviors manifested by the child during the first year (e.g., facial expressions and gestures) are precursors to the lexicon that appears between the second and third year of life (Guidetti, 2002, 2005; Ozcalıskan and Goldin-Meadow, 2005; Callaghan et al., 2011; Cochet and Byrne, 2016; Cochet and Guidetti, 2018; Ger et al., 2018; Moreno and Guidetti, 2018). In particular, Bruner (1983) points out that this continuity is guaranteed through familiar formats or scenarios (standardized models of interaction), or routines (corresponding, for example, to repetitive games), in which the child can identify (and therefore make predictions) based on the regular elements of these scenarios that he already knows, and that the mother or the adult gradually enriches. In this context of interaction, the child also gradually focuses his attention on the variable elements of different situations and on the functions of communication. In other words, he learns the usefulness, purpose, and intentionality of the communicative exchange (Bruner, 1983).

## Communicative Functions in the Context of the Sociopragmatic Interactive Approach

Three communicative functions contextualized in an interactive setting are available to the child during the first two years: Social Interaction (SI), where the intention of the child’s exchange with the adult is to draw attention to himself, to approach, and to obtain physical or social contact (Bruner, 1983; Murray and Trevarthen, 1986; Trevarthen and Aitken Kenneth, 2001; Donelly and Kidd, 2021); Joint Attention (JA), where the intention of the exchange is to share the attention with the interlocutor, toward an object, a person, or a situation (Bruner, 1983; Kinard and Watson, 2015) and Behavior Regulation (BR), where the intention of the child’s exchange is the modification of the behavior of the other, using the adult as an intermediary to obtain from him the help he needs (Bates et al., 1979) or the modification of his own behavior when the adult asks him to do so (Bruner, 1983).

From birth, the child is immersed in social situations in which he is an essential actor, and his intelligence is not only constructed during the relationships he establishes with objects but also with the people around him. Gergely and Csibra (2006) consider that the social environment of a baby is equipped to attract his attention and orient him toward what is important to learn through a communication system that facilitates the transmission of generic knowledge among individuals, as explained in the natural pedagogy theory.

The function of SI is built in these exchanges. In this way, to be successful in the cultural and social world in which he is born, the child must learn the uses of the artifacts, symbols, and social and institutional practices of his contexts. In other words, what makes the child a social and cultural being, similar to adults in the same context, is being able to actively share intentions and attention with other people in collaborative activities (Tomasello et al., 2005; Callaghan et al., 2011; Thommen, 2017; Donelly and Kidd, 2021).

According to sociopragmatic approaches, joint interaction is the basis for the development of the linguistic system (Bruner, 1983; Tomasello, 2003), and researchers have identified the period between 9 and 12 months, as an important stage in its emergence. It has been established that JA is developed around the first year, when children are already communicating with their parents about external objects (Bates et al., 1975, 1979; Bakeman and Adamson, 1984; Carpenter et al., 1998; Kinard and Watson, 2015; Mundy et al., 2016). The development of JA involves two things: on one hand, that children are aware of their environment and, on the other, that they perceive that others are part of the same environment. Carpenter et al. (1998), distinguish three main characteristics of *Joint Attention*. The first is *sharing attention* (appearing from 8 to 9 months) which refers to episodes in which both people focus their attention on the same object, and, during the episode, the baby spontaneously looks at the adult’s face, and then returns to the object. The second characteristic is *directing attention* (around 9–10 months of age), where the child performs an action such as turning the head, pointing, or manipulating an object to retain or redirect the attention of the other person. Finally, *following attention* (from 8 months), where attention is redirected in response to actions such as the direction of the gaze or the pointing of the other person.

On the other hand, in BR the child sees the adult as an intermediary to achieve his goals. In this sense, he regulates the adult’s behavior. In early development, he will do so through gestures, looks, or vocalizations. According to Bates et al. (1979), between 12 and 16 months the child can use the language of adults as an aid in the acquisition and use of gestural patterns. Thus, the child’s understanding of words and gestural production would be related to pointing, a gesture whose objective would be to indicate to the other that he wants something. This linguistic information (in this case received from the adult) influences the gestural performance of the child who imitates some gestural models from adults. Bruner (1983) also relates the function of regulation with the emergence of the request in children (invitations, requests for objects, and requests for help in the actions that he will carry out). According to him, the complexity of the child’s request to the adult implies a regulatory function in the sense that the child not only makes the request, but also controls how he wants it to be satisfied. Bruner adds that this requirement implies not only that the child learns to coordinate his own language with the action requirements of the real world, but also that he learns to do so in culturally and socially accepted ways.

Additionally, as stated by Mundy et al. (2003) and Guidetti and Tourrette (1993/2017), children can play different roles during communicative interactions, namely initiates (*I*), answers (*A*), and maintains (M). Thus, respectively, for each communicative function, the following communicative behaviors can be observed: 3 roles for social interaction (*ISI*, *MSI*, and *ASI*), 3 roles for joint attention (*IJA*, *MJA*, and *AJA*), and 2 roles for behavior regulation (*IBR* and *ABR*).

Those communication functions, however, are not necessarily developed in the same manner across different populations around the world, as they could be affected by environmental constraints. To assess those variables (*SI, JA*, and *BR*), the diversity of the conditions and resources available in the children’s surroundings should be taken into account (Keller and Otto, 2009; Callaghan et al., 2011; Salomo and Liszkowski, 2013; Fawcett and Liszkowski, 2015).

## Socioeconomic Context and Communicative Functions

The socio-economic and cultural backgrounds of children are different around the world and have a substantive influence on language development (Salomo and Liszkowski, 2013; Fawcett and Liszkowski, 2015). Since this linguistic development is a process that is built on the scaffolding provided in the early years by communication functions, it could be inferred that the development of these functions may also be affected by these environments (Keller et al., 2004; Callaghan et al., 2011). However, there is a dearth of research in terms of evaluating this hypothesis, and the studies which are available tend to explore environmental impacts on language development, not on early communicative functions.

One aspect of the social environment that has been related to language development is SES, with findings that support the idea that there are differences across different SES groups, with low-income contexts mostly resulting in detrimental effects on language development. That is, individuals from privileged SES had better indicators related to the appearance of vocabulary. For example, Arriaga et al. (1998) compared linguistic skills through indicators such as size of expressive vocabulary, age of appearance of word combinations, and complexity of expressions. Those indicators were tested by contrasting low-income and middle-income young children matched samples. It was found that scores for the low-income group were strikingly lower on the three key indicators tested.

Similarly, Hoff (2003) showed that children’s rates of productive vocabulary development deviate in relation to differences in language learning experiences derived from their family SES. This study compared two-year old children interacting with mothers from high and medium SES. The results showed a difference between these two groups of children in their productive vocabularies. This difference favored children with high SES whose vocabulary grew to a greater extent compared to the vocabulary of children with medium SES. This difference was explained by the mother’s speech properties that were related to SES.

Additionally, according to Hart and Risley (1995), children living in poverty hear significantly fewer words than their richer peers. This was concluded on the basis of data from 42 American families of diverse socioeconomic origins through monthly hour-long conversations, from the time the children were seven months until the age of three. After following these families for four years, the researchers found that differences in parent-child interactions produced significant discrepancies among children from low-income and high-income families, not only in the children’s knowledge, but also in their abilities and experiences. Follow-up studies showed that these differences in language and interaction experiences have long-lasting effects on a child’s performance later in life.

Fernald et al. (2013) found that significant disparities in vocabulary and language processing efficiency were already evident at 18 months among infants from families with different SES and that at 24 months there was a 6-month gap in critical processing skills for language development between those infants. In a more recent study carried out with 347 Guatemalan children and adolescents, from 6 to 17 years of age, the results showed lower scores in language and attention with respect to the 41.5% of the sample who had a vulnerable background (they came from families with a low socioeconomic level or had had a high exposure to violence) (Ibáñez-Alfonso et al., 2021). According to Locke et al. (2002) and Ginsborg (2006), many low-income children already have a noticeable delay in the development of their language ability by the time they turn three years old. This suggests that more attention should be paid to the communication experiences of children during the first three years of life. To a lesser extent, early communicative experiences have also been related to the socioeconomic context (Tamis-LeMonda et al., 2014; Barbu et al., 2015; Hirsh-Pasek et al., 2015) and have been studied from a sociopragmatic perspective that deems social interaction as a central component in the development of communication and language (Donelly and Kidd, 2021).

Nowadays it is known that the development of socio-communication skills in children depends on socialization and childcare practices (Gaffan et al., 2010). Recently, more research has been conducted comparing children aged 0 to 3 years living in different socioeconomic contexts (Bornstein et al.,2015a,b). Especially in low-income countries, socialization and childcare practices are determined by social and economic conditions, as well as the working conditions of mothers and/or caregivers (Bornstein et al.,2015a,b).

Therefore, it could be expected that children who grow up in contexts with vast social differences would also show significant variation in the development of their social skills and communication functions. Studies linking the behavior regulation function and socioeconomic context from a pragmatic perspective are scarce. Those that are available tend to be associated with parenting practices and behavior problems, rather than with communication development. These studies are also carried out with children older than 3 years. For example, a study developed in Norway by Størksen et al. (2015) supports the idea that children’s behavior regulation may be influenced by their contexts and cultures, including their parents’ socioeconomic status.

On the other hand, Pisani et al. (2018) conducted a study with 204 Brazilian mothers of children from 3 to 8 years old. The results revealed three latent parenting practices: emotional and behavioral regulation, communication, and positive discipline. Lower socioeconomic status was directly related to higher levels of internalization of child behavior problems and more negative parenting practices in the domains of positive communication and discipline. Although mothers’ emotional and behavioral regulation was not related to socioeconomic status, it was a negative predictor of children’s behavior problems. Overall, these studies show, on the one hand, that there is a relationship between socioeconomic status and behavior regulation, and on the other hand, that further research is needed concerning the regulation of behavior at an early age.

Moreover, studies that relate the socioeconomic context and joint attention function specifically in children under three years of age are also scarce. Gaffan et al. (2010) evaluated fifty-nine healthy babies who were filmed with their mothers and with a researcher at two, four, six, and nine months in face-to-face games and at six and nine months in games with toys. Specifically, the child attention request and the percentage of time in shared attention was evaluated. None of the demographic, cognitive, or psychiatric variables measured had a significant effect on joint attention for the nine-month children, alone or in combination with other variables, in line with previous studies that have reported weak and inconsistent associations for joint attention and other variables (Goldsmith and Rogoff, 1997; Carpenter et al., 1998, 2002; Mundy et al., 2003). It is becoming increasingly evident that joint attention appears to be a common core communicative skill that develops in children around the world (Callaghan et al., 2011; Liszkowski et al., 2012).

The innate and adaptive explanation of the origin of cognition and joint attention has been contrasted with the cultural explanation. Authors such as Callaghan et al. (2011) explain that the triadic interaction (joint attention) around objects among parents and their young children is not universal in all cultures during the first 2 years of life, at least not in the prototypical form or level at which it occurs and is typically characterized in the scientific literature. However, other studies have tacitly assumed the universality of co-attention processes in middle-class western parents and children, e.g., Bruner (1983) and Tomasello et al. (1999).

## Cultural Context and Communicative Functions

Although research on the development of communicative experiences in early childhood has increased in recent years, these studies have mostly focused on exploring, for example, how communicative experiences are influenced by the cultural context (Liszkowski and Brown, 2007; Callaghan et al., 2011; Liszkowski, 2011). Referring particularly to the pointing gesture in children (one of the most characteristic indices of prelinguistic communication and which is key in the development of joint attention), Liszkowski et al. (2012) developed an investigation that compared 7 different cultures. The results of their study showed that the gesture of pointing emerged in all cultures within the same age range that had previously been established for American samples. Even the frequency of the baby’s pointing did not differ between cultures. According to Liszkowski et al. (2012), these results refute previous research that had questioned the universality of prelinguistic communication skills, alluding to vast cultural differences in socialization practices and the role of social interaction in development. For example, in the case of joint attention, most studies agree that it develops in the same manner in all cultures (Liszkowski et al., 2012), and that it is in fact one of the vital cognitive and communicative functions developed by the human brain (Mundy, 2016; Nyström et al., 2019). These results, furthermore, bring into question the idea of whether the development of communicative functions could also be universal.

## Core Hypothesis of This Work

This research, therefore, assumes that if a communicative function is universal, its development should not be affected by the social environment including SES; but, considering the previously mentioned studies, it can be argued that this could be the case for Joint Attention but not for Social Interaction and Behavior Regulation. This central idea constitutes the core hypothesis of this research. Therefore, as Social Interaction and Behavior Regulation seem to depend more on the context and care practices received by children in their first three years than Joint Attention, when examining these variables in samples with diverse SES, it is expected that the socioeconomic environment would influence the first two of them (SI and BR) but not the last one (JA). As known, it is with their parents or caregivers that the child normally establishes the first social interactions, but it is clear that, if the parents do not have the appropriate social and economic conditions, these interactions could be diminished or not stimulated in the child (Conger and Donnellan, 2007; Leventhal et al., 2015).

## Contextualizing the Research Hypothesis: The Development of Communicative Functions in Colombia

The foregoing discussion has particular salience in the context of countries with sharp inequalities and lack of social mobility, where the opportunities to exercise the acquired interactive skills are more limited. A suitable place to test the stated hypothesis is Colombia (more specifically the Caribbean coast). Colombia is characterized by a highly unequal society, where families live in very different socioeconomic conditions, ranging from very wealthy to very poor neighborhoods; and where the difference between the wealthy and the poor is very marked.

Efforts to measure and situate these differences have been made by several organizations, for example, according to the Organization for Economic Cooperation and Development (OECD, 2018), Colombia ranks 65 out of the 82 countries evaluated, where families need around 12 generations to change their SES. Indeed, when the results of the last Large Integrated Household Survey in Colombia (GEIH) are analyzed, the percentage of people classified as poor with respect to the national population is 35.7%, and the percentage of people classified as living in extreme poverty with respect to the total national population is 9.6% (DANE, 2021d). In the latest UNDP (2020), although Colombia is classified high in terms of the human development index (0.767), extreme social and economic distances were observed. For instance, the World Bank reported that Colombia’s Gini coefficient was 0.53 in 2019. Further, according to a report by the Economic Commission for Latin America and the Caribbean (ECLAC), the level of extreme poverty in Colombia increased to 14.3% in 2020 due to the COVID-19 crisis.

### Understanding Socioeconomic Strata in Colombia

To address the inequality gap, in 1994 the Colombian government approved the National Utilities Law (Ley 142 de 1994, Ley de servicios públicos. See art. 102) that allowed for the classification of households according to their housing conditions, with a tool known in the country as Socioeconomic Strata (*Estrato Socioeconómico*) (DANE, 2021a). Specifically, housing units were assigned a number from 1 to 6, to cluster them based on their physical and structural features and the conditions of the neighborhood where they were located. The goal of this classification is to tax utilities in such a way that more affluent households subsidize services for families in poverty. In this sense, low strata households receive benefits from the government in terms of reduced monthly bills.

In this classification, strata 1, 2, and 3, are the lowest, with 1 being very low and equal to extreme poverty, 2 being low, and 3 middle/low. These strata are assigned to housing units whose inhabitants are considered to be in situations of poverty, with houses that have structural problems such as lack of windows and walls, built using precarious materials (sand, clay, wattle, wooden frames, and pallets), that may lack access to public utilities (water, gas, and electricity), and that are located in neighborhoods with infrastructural problems, such as lack of pavements, no access to public transportation, and located on geologically unstable land (DANE, 2021c). Stratum 4 is considered a middle classification for the quality of housing conditions. Households in this stratum have access to public utilities and have adequate structural characteristics; they are located in urbanized neighborhoods and do not receive benefits from the government in their utility bills, however, they are not subjected to a higher tax rate to subsidize the lower strata. Lastly, strata 5 and 6 (middle/high and high, respectively) have the highest quality housing conditions, both structurally and locationally, being situated in safe neighborhoods that usually have access to parks, recreational centers, malls, and so on. Their residents are taxed highly, and their utility bills comprise both their consumption and an additional percentage that is charged to subsidize strata 1, 2, and 3 (DANE, 2021c).

### Mapping SES and Socioeconomic Strata

Although income is often related to housing conditions, it is not straightforward to map SES to strata. This is because strata only consider structural, urbanistic, and construction features of households and do not take into account other indexes that are usually measured to assess overall quality of life in terms of SES, as SES determines ability to access goods, resources, services, and safety that are essential to human development, e.g., access to private schools, music classes, organized sport, and technology (Duncan et al., 2015). This represents a disparity between what is understood as poor in a developed country observed under the SES paradigm versus what can be considered as poor in Colombia under the strata paradigm. However, there is some common ground in both definitions, for example high SES and high strata neighborhoods are often safe and guarded. In contrast, low SES and low strata translate to living conditions with a shortage of resources to guarantee food security, housing and overall safety, and high levels of stress in the communities (Duncan et al., 2015). In this sense, and for the purpose of comparison, some equivalences to the classifications are identified to define SES levels in Colombia, considering strata or housing conditions, by the Colombian National Administrative Department of Statistics (DANE, 2021c). These are as follows: Very Low SES or extreme poverty (stratum 1), Low SES (strata 2 and 3), Middle SES (stratum 4), and High SES (strata 5 and 6). In this research the DANE equivalence is used to characterize the sample.

### Extreme Poverty and Very Low SES in Colombia: The Case of the Wayuú Ethnic Group

Extreme poverty, Very Low SES, is usually observed in Colombian ethnic settlements. One of the most predominant ethnic groups on the Colombian Caribbean Coast is the Wayuú tribe. According to Ariza et al. (2017), the Wayuú are the largest indigenous group in the region and in Colombia, although other indigenous groups also inhabit the same region. Due to its climatic and topographic conditions, this region is considered a desert, empty, arid, and hostile territory. Access to public services is very limited and access to drinking water and basic sanitation are inadequate. As Russell et al. (2020) describe, “only about 12% of the Wayuú live in urban centers, the vast majority live in Wayuú communities that are generally rural, very dispersed, and difficult to access. Many live in small family group settlements, called *rancherías*, which may consist of only a few houses. […] The houses are usually made of wood with clay plaster and contain dirt floors. Communities are often physically isolated, even from one another, with almost no paved roads or public transportation […].” This study also found that all the Wayuú children (aged 0 to 5 years) met the criteria for either moderate and severe malnutrition, stunting, or underweight.

### The Importance of Studying Communicative Functions Development in Context

Social communication skills are a key aspect of early childhood development. They influence not only the development of relational and affective competence (Gaffan et al., 2010) and social cognition (De Jaegher et al., 2010), but also the later development of language (Ozcalıskan and Goldin-Meadow, 2005; Callaghan et al., 2011; Ger et al., 2018). In this sense, it is considered that the environment may offer opportunities to exercise the interactive skills that are developed in the first two years of life and that provide a basis for all ensuing social and communication developments. But research has also shown that when the environment involves a range of negative exposures during early childhood that include situations such as abuse, stimuli deprivation, neglect, chronic poverty, among others forms of adversity (Burghy et al., 2012; Cohen et al., 2013), children have higher probabilities of developing physical and mental health problems, including cognitive, memory, attention, implicit and explicit learning processes and language development (McLaughlin et al., 2014; Heleniak et al., 2016; McGrath et al., 2017; McLaughlin and Sheridan, 2017). Thus, it could be argued that such populations as the ones described in Colombia, which exhibit high levels of social inequality, early childhood adversity, and even malnutrition could be subject to communicative skill deficits due to their disadvantageous conditions.

### Testing the Core Hypothesis in Colombian Samples

As the results presented in the literature reviewed pose the question of whether different SES groups could possibly exhibit differential development in their communicative functions (*SI*, *JA*, and *BR*), this research focuses on testing communicative functions across different children samples from diverse SES, in Colombia, including children from a Wayuú settlement, as they represent an interesting testing ground for the hypothesis considering that not only do they have Very Low SES, but also differ from their peers in their ethnic and cultural backgrounds. To the best of our knowledge, this is the first research to directly assess this hypothesis in the Colombian context using the ECSP scale developed by Guidetti and Tourrette (1993/2017).

## Materials and Methods

### Participants

This research takes place on the Colombian Caribbean Coast. The participants were 36 Colombian children (21 boys and 15 girls), 25 months old on average (*SD* = 3.99) and classified according to the SES of their families (see Table 1, detailing the mean age and SD of age per SES group as well as the gender distribution per subgroup). Their classification corresponds to their family’s SES, in accordance with the DANE guidelines, as described in the section *“*Introduction.” No statistically significant differences were found among SES groups’ average age, this can also be observed from the overlapping confidence intervals. Sample subjects were selected from across several SES to have cases spanning representative social and economic distances, in consonance with the main concern of this work. After accounting for eligibility and exclusion criteria, the final sample of 36 subjects was distributed evenly, as follows: 12 Very Low SES, 12 Low SES, and 12 High SES. This reflects our research goal of screening and assessing for opposite and extreme SES differences (such as High vs. Low SES and Very Low SES), in concordance with similar research assessing SES, as presented in the Introduction subsection “Socioeconomic context and communicative functions.”

**TABLE 1:** Sample subjects average age (in months) and standard deviation by socioeconomic status (SES).

	Mean age	Standard deviation
**Very Low SES** *n* = 12 (9 boys, 3 girls)	23,25	4,41
**Low SES** *n* = 12 (6 boys, 6 girls)	25,91	2,79
**High SES** *n* = 12 (6 boys, 6 girls)	25,32	4,09
**Total sample** *n* = 36	24.83	3.99

Very Low SES children were selected from a rural Wayuú-only non-profit childcare center, as they exhibited both an extreme difference in SES from their High SES peers and also in ethnic/cultural differences. Broadly, being a member of an ethnic group does not necessarily translate into belonging to a particular SES group and *vice versa.* However, the Wayuú are remarkably marginalized in Colombia, and being a member of this ethnic group usually results in living in extremely impoverished housing conditions (stratum 1). For the purpose of this research, and according to Colombian law, the Wayuu sample is considered as Very Low SES (DANE,2021b,c).

### Materials

In this research the main tool used was the ECSP [the acronym for Echelle d’évaluation de la Communication Sociale Précoce – the standardized and adapted French version of the American Early Social Communication Scales (Seibert and Hogan, 1982), by Guidetti and Tourrette (1993/2017)]. The ECSP scale is one of the most promising tools currently available for the evaluation of early social communication in all its complexity. It consists of a methodological instruction manual that includes administration guidelines, instructions for evaluation and score and scale sheet usage, and a list of playful situations that allow interaction with the child and the toys or objects used by them.

The ECSP is of special interest because it allows for evaluating the development of communication from the first months of life (0–30 months) and assesses interactive and prelinguistic communication. Moreover, its reliability has been confirmed by studies with large samples and it clearly distinguishes between typical and atypical development. In France, it is the tool recommended by the National Health Authority and the French Federation of Psychiatry to evaluate communication in subjects who are autistic or deaf. It is usually applied to children between 3 and 30 months, but since its upper limit of application is the point at which the child begins to combine words, it can also be used with older children who are atypical in development. This French scale has also been translated and validated into Italian (Molina et al., 2016).

For this work, the ECSP was translated from French to Spanish and adapted for the Colombian context (Moreno and Morán, ECSP-E, in prep.). To do so, a process of linguistic and cultural equivalence to Spanish was carried out. The linguistic equivalence process involved the following. First, a native bilingual (French-Spanish) Colombian researcher translated the instructions and the answer sheet (where the expected communicative behaviors of the child were to be recorded) from French to Spanish. Then a second independent researcher (bilingual French – Spanish) translated the Spanish version to French. Finally, the original version and the back-translated version were compared to identify and correct differences (Peña, 2007). To guarantee the process of cultural equivalence, native evaluators, who knew the local context and the conventional manners in place, were enrolled to validate the pertinence of the Spanish translation. In line with this rationale, storybooks and local nursery rhymes were adapted to the local context during the assessment.

**FIGURE 1:**
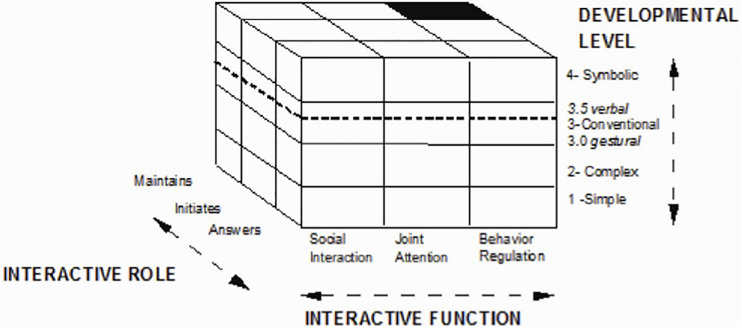
ECSP’s Developmental levels and interactive roles and communicative functions.

The ECSP consists of 23 interactive and playful situations that allow for eliciting and observing 108 possible expected communicative behaviors indexed in three communicative functions (SI, JA, and BR) and arranged according to their developmental complexity from simple to complex. The design of the activities allows for evaluating several communicative functions at once, through a series of items. The scale includes 8 sets of items in which it is possible to obtain a score and an optimal level. These sets contain the grouping of expected communicative behaviors by communicative function (IS, JA, and BR), role played by the child (initiates I, answers A, and maintains M), and their developmental levels. Those levels are arranged hierarchically as follows: 1. simple, 2. complex, 3. conventional (3.0 conventional gestural and 3.5 conventional verbal), and 4.0 symbolic (see Figure 1 below). Scores for each communication function can be mapped to developmental ages of children, so for typical children scores should also match their chronological age. Thus, for example, for each level, there will be 8 items that will include communicative behaviors for each function (SI, JA, and BR) and for the role played by the child in response to the interaction with the adult: (I), (M), and (A). In sum, at each level there will be 3 sets of items for social interaction (ISI, MSI, and ASI), 3 sets for joint attention (IJA, MJA, and AJA), and for behavior regulation there will be 2 sets of items (IBR and ABR). In this last function, the maintenance of behavior regulation is not evaluated.

Regarding the hierarchy, each optimal level can be interpreted in terms of development as follows:

1. simple: 0–2 months – This level represents the beginning of intentional activity in the child’s interaction with others.
2. complex: 2–6 months – In this level the child begins to participate in social games and can differentiate people; however, he does not coordinate actions with objects or with others.
3. conventional: 7–24 months – This level marks a step-change in the communicative skills of the child; he learns gestural and verbal conventions, and learns how to use objects to draw attention to himself or to use the interlocutor to obtain something.

3.0 Conventional gestural: 7–16 months – The child learns gestural conventions and uses them.
3.5. Conventional verbal: 16–24 months – The child uses isolated words in the presence of objects (objects’ names and action verbs) either to replace or to complement gestures. This level marks verbal progress in communication skill.
Note: A child’s placement in levels 3.0 and 3.5, although it represents a great step forward in his communication skills, also means that h is understanding of situations is still highly dependent on the context.

4 symbolic: 25–30 months – This level represents the emergence of symbolic functions and marks decisive progress in the evolution of the child’s communicative capabilities. The child is capable of anticipation and initiation, and this allows him to understand words out of context or with little context. The child combines words to maintain an interaction and to ask for or exchange information with others about his surroundings. Their social games are then transformed by incorporating the symbolic dimension (Guidetti and Tourrette,1993/2017).

### Procedures

#### Sample Subject Recruitment

To recruit sample subjects, invitation letters were sent to different non-profit early childhood care centers, as well as to private day care centers and directly to families from selected neighborhoods. Government non-profit early childhood care centers for low class citizens, that provide initial education, care, nutrition, and medical care for children under 5 years of age (Instituto Colombiano de Bienestar Familiar, ICBF, 2021) are in place in the country. On the other hand, middle- and high-class children receive nutrition and care mostly at their homes or attend private day care centers; so, invitations were sent to parents from all these diverse setups to obtain a sample representative of different childcare conditions present in the country for diverse SES.

The invitation letter explained the intent and scope of the study and the evaluation procedures and protocols to be followed during the evaluations of the children. 55 parents or caregivers agreed to participate in the study and were sent an informed consent form and a questionnaire eliciting information about the children and their developmental medical history (for example, pre, peri, and postnatal history, diagnosis of clinical and neurodevelopmental diseases), which allowed for the selection of 43 participants that met the following inclusion criteria: *(i)* that the children were between 17 and 24 months of age *(ii)* that they were in good health (confirmed by their parents, caregivers, or their pediatrician), and *(iii)* that their families were classified in one of the three contrasting socioeconomic levels (very low, low, and high) of interest. Later, an appointment was scheduled to undertake the evaluation session. After the recording sessions, exclusion criteria were applied which lead to a total of 36 recorded sessions to be evaluated. Exclusion criteria for recordings are detailed in the next subsection *“Evaluation procedure.”*

#### Evaluation Procedure

All selected children were individually evaluated during one approximately 40-min session that took place either at one of the non-profit early childhood care centers or at the University’s Gesell chamber for sample subjects from private daycare centers or selected families. Evaluation rooms were isolated from outside noise and surrounding distractions and were also adapted in advance to resemble a game room as much as possible, so that the child felt safe to play with the evaluators, who were native psychologists trained in the application of the scale, and who always addressed the children in Spanish. All evaluations were recorded, for *a posteriori* evaluation and scoring, and performed in the presence of one parent or caregiver.

All sessions followed an evaluation protocol. First, the child entered the evaluation room accompanied by his parent or caregiver, who had previously been instructed not to intervene during the session. In the room there was also a person in charge of filming the session; they had also been instructed not to intervene. After the child had enough time to become familiar with his surroundings, a trained evaluator entered the room silently and greeted the child; this step, interactive situation #1, marked the beginning of the evaluation session. Interactive situations followed a specific order but were flexible enough that they could be adapted to the child’s responses, as on occasion the child could exhibit communicative behaviors that changed the course of the planned progression. These could be used by the evaluator to hold the child’s attention and take advantage of it to channel the direction of the session to guarantee the observation of behaviors of interest. Interactions were structured to follow a sequence from the evaluator’s entrance, the introduction of social objects, mechanical or other attractive age-appropriate toys, puppets, and materials, such as stories, tales, picture books, singing, and nursery rhymes, to the evaluator parting after a total of 23 playful situations. However, if a child exhibited a behavior indicative of discomfort, the evaluation was concluded; such behaviors include for example consistent crying and overt tiredness. Those sessions were excluded from the analysis, as well as others that met the following exclusion criteria: bad quality sample recordings, behaviors not visible in the recordings after undertaking the evaluation session due to subject positioning.

Object introduction also followed a setting and presentation order. Toys were saved and put together in a closed box located at a safe distance that allowed the evaluator to oversee presenting the objects. The evaluator showed the toys to the children according to the situation and then stored them back in the box at the culmination of the staged situation, making way to proceed with the next situation and its respective objects. As the evaluation approach is based on an interactive paradigm, all playful situations used for testing are interactive and require the total investment of the evaluator in the activities while also engaging the children.

#### Data Treatment

Coding, processing, and data analysis of the resulting 36 recorded sessions, obtained after applying inclusion/exclusion criteria, were performed *a posteriori*. For each situation, all the communicative behaviors exhibited by the child were marked on an answer sheet. The answer sheet included the item number of the situation in the test, its level of development, and its content. The number of boxes marked for each item corresponds to the number of times the behavior is required for the item to be considered as achieved. The achievements are then transferred to the score sheet to assess the performance at developmental levels, in each of the 8 sets of the three communication functions and for the entire test.

The score was calculated based on 5 points per level (5 levels), which allows children to obtain a maximum of 25 points for each series of items (8 series), which is equivalent to a total of 75 points maximum for the series of social interaction and joint attention items, 50 points for the series of behavior regulation items, and 200 points for the entire scale. Obtaining all the elements of the same level allows the child to accredit previous levels. Beyond that, each item obtained allows a credit for a certain number of points that varies depending on the number of items present in the level. The optimal level corresponds to the highest level in which an item was obtained for each series of items, for each communicative function, and for the test (Guidetti and Tourrette,1993/2017).

In summary, scores for the child’s development level were calculated through the following evaluation indexes:

• **Optimal level** in each series (it is the highest level where an item was obtained), for each scale, and for the whole test (the highest level among all the levels reached). Calculated for
  • Series of items per role per communication function (ISI, MSI, ASI, IJA, MJA, AJA, IBR, and ABR): from levels 1 to 4
  • Communicative function scale (SI, JA, and BR): from levels 1 to 4
  • Test as a whole: from levels 1 to 4
• **Score:** for each series of items, for each scale, and for the test as a whole, namely
  • Series of items per role per communication function (ISI, MSI, ASI, IJA, MJA, AJA, IBR, and ABR): From 1 to 25 for each one.
  • Communicative function scale (SI, JA, BR): from 1 to 75 for SI and JA, and 1 to 50 for BR
  • Test score as a whole: from 1 to 200

Finally, the scale manual indicates that the interpretations of the results can be analyzed in terms of developmental levels or scores, without the need to assign ages of communicative development, to establish a communicative profile of the children (Guidetti and Tourrette,1993/2017). Development levels are not only used to identify if communicative milestones are reached, but also determine in detail where the child is at regarding the role in the interaction for each function and the total scale. In this sense, interpretation by levels will be used to evaluate the impact of the SES on the development of communicative functions in sample groups.

#### Intercoder Reliability

Researchers involved in this work were trained in ECSP coding, evaluation application, situation configuration, assessment, scoring, session recording, and the theoretical context of the ECSP. To guarantee intercoder reliability, videos were always observed by at least two teams of trained psychologists, who worked independently on coding using the answer sheet, thus double-coding. After coding and double-coding, the teams regrouped to reach consensus on their impressions. Protocol dictated that in the case of incongruencies, videos must be re-watched to verify and register a final agreement. After consensus, behaviors were graded on a score sheet, using a point system that allowed for computing the scores and optimal levels for items series by role, communicative function, and total test scores.

The coding was carried out using the answer sheet where observations corresponding to expected possible communicative behaviors were registered with 1 or 0, whether the communicative behavior had been exhibited or not by the child, respectively. Reliability was established by comparing coders’ transcribed and encoded communicative behaviors displayed by 9 participants (25% of the sample, who were selected at random), resulting in 2277 binary observations. Following the recommendations of McHugh (2012), an intercoder percentage of agreement was computed and it was found to be suitably high, 85.59%.

#### Experimental Design

A between subject analysis design was developed for this work, considering SES as the independent study variable. Other variables such as age and gender were not considered in the scope of this research. Considering the small sample size and the small effect size of gender on score differences, this variable was not considered in this study. As for the ages, its potential effect on the results was minimized by recruiting sample subject children that were around the same average age. This is, to determine if SES was a differential factor in scores, and considering that, given the milestones of development, younger children normally score lower than older ones, children were selected trying to homogenize for age.

The SES variable was controlled in sample subjects by grouping participants in the three SES of interest, so that 12 children were assigned to each group, in the following categories: Very Low, Low, and High. Each participant was evaluated individually and for each case the procedure was consistent. Consequently, there was statistical independence between SES sample groups. Dependent variables of interest that were evaluated in this work are the child’s communicative function (SI, JA, and BR), evaluated through indexes such as optimal level and score. Communication functions variables are described in this study, according to the ECSP scale (Guidetti and Tourrette,1993/2017), as follows:

##### Social Interaction (SI)

Assesses the development of a child’s abilities to interact with an adult and participate in playful exchanges with him. It is the interaction itself that is central and not the objects or events that sustain this interaction. These exchanges were not imperative or related to an adult’s request. They take place in gestural and verbal social games, imitation games, or object-sharing games between the child and the adult.

##### Joint Attention (JA)

Assesses the development of a child’s skills to establish shared attention on the same object, person, situation, or subject. Joint attention differs from social interaction in that it is the objects or situations that are subjected to attention and not the adult-child interaction. Furthermore, there is no intention to act on the object, only to look at it together. As this function develops, it may include an exchange of information about the properties and characteristics of the object or situation.

##### Behavioral Regulation (BR)

Assesses the development of the child’s ability to modify or influence a behavior, either his own or that of the adult. This results from the fact that to access what he wants or needs, the child requires the help or cooperation of an adult. Attempts to regulate the behavior of his interlocutor are often imperative or didactic in nature, can be (but are not necessarily) prohibitions as they are executed firmly but not harshly. In the first levels of development (see Figure 1), this series of items assesses the child’s ability to guide and inhibit his actions based on external indications, as well as the growing awareness of the child that his behavior is controlled by forces external to him. At the highest levels of development (see Figure 1), what varies is the breadth of the gestural and contextual cues that the child needs to understand the verbal instructions of adults (Guidetti and Tourrette,1993/2017).

#### Data Analysis

For data analysis, total raw scores resulting from the tabulation of the items related to joint attention, behavior regulation, and social interaction were considered. Total raw scores and levels for each function by role of the child in the interaction (initiation, maintenance, and answer) and by optimal level of development were analyzed. Once the data collection was completed, they were tabulated and analyzed through descriptive and inferential (categorial and non-parametric) statistics using the SPSS software package (version 22, IBM statistics).

## Results

In this section, the results of statistical analysis are presented concerning the assessment of dependent variables used in this study. Scores and optimal levels were measured for items series by role, communicative function, and total test scores. Developmental communicative levels are ordinal variables that allow establishing profiles for children’s communicative skills, while scores have measurements across a continuous scale that allow for deeper non-parametric comparisons of the tested items.

Considering the small sample size and that the scores were not normally distributed, the Kruskal-Wallis H test was selected for non-parametric analysis. This method is appropriate for more than two independent groups and is capable of assessing statistically significant differences between them. The effect size over paired groups that tested significantly different was calculated through a *post hoc* test. Hereafter, the following conventions are used: ***M*** for mean, ***SD*** for standard deviation, ***MR*** for middle range, ***X*^2^** for chi square,***Me*** for median, and ***p*** for p-value.

### Descriptive Statistics

#### General Descriptive Statistics for Raw Scores

The main descriptive statistics for all score measurements are presented in Table 2. This table shows the mean of the item series raw scores per communicative function obtained by the participants, the standard deviation, the value of the range (difference between the largest and smallest values of the scores), skewness, and kurtosis. As stated before, the score was calculated based on 5 points per level (5 levels), which allows children to obtain a maximum of 25 points for each series of items (8 series), which is equivalent to a total of 75 maximum points for the series of social interaction and joint attention items, 50 points for the series of behavior regulation items, and 200 points for the entire scale.

**TABLE 2:** Descriptive statistics.

	*M*	*SD*	Range value	Skewness	Kurtosis
SI	25.150	21.753	69.42	0.688	–0.804
JA	38.115	15.647	69.67	–0.017	0.134
BR	20.798	13.131	43.75	–0.392	–1.039
ASI	11.326	9.180	25.00	0.211	–1.550
ISI	7.458	7.338	21.67	1.124	–0.431
MSI	6.365	9.327	25.00	1.144	–0.529
AJA	15.638	4.702	23.00	–2.007	5.558
IJA	9.004	7.198	25.00	0.800	0.018
MJA	13.472	6.151	23.33	–0.373	–0.640
ABR	9.444	8.652	25.00	0.143	–1.597
IBR	11.354	6.277	25.00	–0.035	–0.568

*Item series scores per communicative functions (SI, social interaction; JA, joint attention; BR, Behavior regulation); Interactive role [Answers (A), initiates (I), maintains (M)].*

It can be observed from the skewness and kurtosis values in Table 2 that the data were not normally distributed, thus necessitating the use of a non-parametric method of inferential analysis.

### Non-parametric Analysis

#### Socioeconomic Status and Communicative Functions

In this section, results for the Kruskall-Wallis test are shown for the three communicative functions measured though raw scores: *SI*, *JA*, and *BR*, between the groups of interest (very low SES, low SES, and high SES), see Table 3.

**TABLE 3:** Kruskal-Wallis statistics for communicative functions in Social Interaction (SI), Joint Attention (JA), Behavior Regulation (BR), and Total Score (TS) by socioeconomic status (SES).

	Kruskal-Wallis Test				
	SES	*n* = 36 (12*3 SES)	*X*^2^	*p*	*MR*
SI			9.575	0.008*	
	Very Low				10.92
	Low				21.25
	High				23.33
JA			3.001	0.223	
	Very Low				-
	Low				-
	High				-
BR			5.594	0.061	
	Very Low				-
	Low				-
	High				-
TS			6.955	0.031*	
	Very low				12.04
	Low				20.79
	High				22.67

*p < *0.05.*

Kruskall-Wallis results allow validating statistically significant differences among groups. Table 3 shows that the null hypothesis of no significant difference could only be rejected with respect to SI, and not for JA or for BR. In this function, a *post hoc* Mann-Whitney test showed that the differences were found particularly between the Very Low SES group and the High SES group (*p* = 0.004); and between the Very Low SES group and Low SES group (*p* = 0.016). There were no differences between the Low and High SES groups (*p* = 0.628). Similarly, the paired groups Very Low SES and Low SES, and Very Low SES and High SES were further validated as significantly different when accounting for the Total Score (TS) using a *post hoc* Mann-Whitney (*p* = 0.042 and *p* = 0.013, respectively).

#### Socioeconomic Status and Child Roles per Communicative Function

Given that children can play three roles in the situations of interaction with the adult (answers, initiates, and maintains) in the three communicative functions (except for Behavior Regulation, where the child can only play two roles: answers and initiates), a second analysis detailing items series by role per communicative function was carried out, as shown in Table 4.

**TABLE 4:** Kruskal-Wallis statistics for series of items of Joint Attention (JA), Social Interaction (SI), and Behavior Regulation (BR), according to interactive role of child: A (answers); I (initiates) and M (maintains), by socioeconomic status (SES).

	Kruskal-Wallis Test				
	SES	*n* = 36 (12*3 SES)	*X*^2^	*p*	*MR*
ASI			10.125	0.006*	
	Very Low				11.13
	Low				19.83
	High				24.54
ISI			5.686	0.058	
	Very Low				13.42
	Low				18.58
	High				23.50
MSI					
	Very Low		3.701	0.157	14.96
	Low				22.75
	High				17.79
AJA					
	Very Low		2.626	0.269	14.63
	Low				9.71
	High				21.17
IJA			1.591	0.451	
	Very Low				15.88
	Low				18.38
	High				21.25
MJA			2.877	0.237	
	Very Low				14.38
	Low				20.04
	High				21.08
ABR					
	Very Low		9.706	0.008*	11.00
	Low				22.46
	High				22.04
IBR					
	Very Low		1.068	0.586	16.29
	Low				18.71
	High				20.50

*p < *0.05.*

As seen in Table 4, significant differences were found between the three groups in the series Answer to Social Interaction (ASI) and Answer to Behavior Regulation (ABR). A *post hoc* Mann-Whitney test showed that the differences were found for the ASI series between the Very Low SES group and Low SES group (*p* = 0.042); and Very Low SES and the High SES group (*p* = 0.002). For the ABR series, differences were also found between the Very Low SES and the High groups (*p* = 0.008); and between Very Low SES and Low SES groups (*p* = 0.006). It is important to recall that in the first general analysis carried out on the three communicative functions (Table 3), the results did not show significant differences between the groups with respect to the Behavior Regulation function (*p* = 0.061), however, this second detailed analysis by series of items evidenced that the groups differ in terms of the response to behavior regulation as shown in Table 4 and also that results for ASI and ABR series showed significant differences between the Very Low SES group and the other two groups (Low SES and High SES). These last two did not show significant differences between each other.

#### Socioeconomic Status and Communication Development Levels

A third analysis was carried out to assess differences between the groups (Very Low SES, Low SES, and High SES) in terms of the level of development in communicative functions. These levels, as explained in the section“Materials section,” and shown in Figure 1, correspond to simple (level 1), complex (level 2), conventional gestural (level 3.0), conventional verbal (level 3.5), and symbolic (level 4). Table 5 presents results of the non-parametric Kruskall-Wallis tests for levels of development by communicative function. Therein, no significant differences are discernable between the socioeconomic groups in terms of the level of development for the communicative functions of SI and BR, but not for the level of development of JA. A *post hoc* Mann-Whitney test showed that the differences were found particularly between the Very Low SES group and the High SES group (*p* = 0.001) for the level of development in social interaction. On the other hand, for the level of development in the regulation of behavior, significant differences were found between the Very Low SES and Low SES groups (*p* = 0.004).

**TABLE 5:** Kruskal-Wallis statistics for optimal level of development by communicative function (SIL, social interaction level; JAL, joint attention level; BRL, behavior regulation level) and Final optimal Level (FOL) by socioeconomic status (SES).

	Kruskal-Wallis					
	SES	*n* = 36 (12*3 SES)	*X*^2^	*P*	*M*_*e*_	*MR*
SIL			11.357	0.003*		
	Very Low				3.0	11.25
	Low				3.5	19.88
	High				4	25.17
JAL			4.259	0.019		
	Very Low					-
	Low					-
	High					-
BRL						
	Very Low		8.815	0.007*	2	12.29
	Low				4	23.96
	High				3.5	19.25
FOL						
	Very Low		10.059	0.007*	3.5	11.50
	Low				4	20.75
	High				4	23.25

*p < *0.05.*

Regarding the level of development of social interaction as a communicative function, children in the lowest context (Very low SES) obtained the lowest level of development: level 3.0, conventional gestural, with respect to their peers in Low SES and High SES groups, who were located at levels 3.5 and 4, respectively.

With respect to the communicative function of behavior regulation, it is important to highlight that the Low SES group obtained the highest level of development (level 4, symbolic level); this was the only situation in which the High SES group did not score the highest level. Statistically significant differences were found between the Very Low and Low SES groups, with a *post hoc* Mann-Whitney *p*-value = 0.004.

Finally, Table 5 shows that significant differences for the Final Optimal Level, FOL (which includes the three communicative functions), among different social groups (*p* = 0.007) were found through the Kruskall-Wallis test. A *post hoc* Mann-Whitney test showed that differences were found particularly between the Very Low SES group and the High SES group (*p* = 0.003) and between the Very Low SES and Low SES groups (*p* = 0.018).

## Discussion

The goal of this study was to investigate the influence of the socioeconomic context on the development of communication and language in young children. In particular, this study examined the level of development of three communicative functions: SI, JA, and BR. As far as we know, this is the first study to explore the development of these early communication skills in children who live in different social contexts in Colombia; also, using a scale that specifically measures this development and is based on sociopragmatic approach of development whose core is the interaction.

Three main findings were obtained. First, social context, in terms of SES, influences the level of development of two early communication functions: Social Interaction and Behavior Regulation. Second, it was found that Joint Attention was not influenced by SES. This result is in fact very reassuring as this function is key both in the development of communication and language and social cognition. Third, for functions with statistically significant differences, the Very Low SES sample group always had the lower rank of development optimal level, however, the Low SES sample group did not display delays in their optimal level results. Each of these findings will be discussed in detail in the following subsections.

### SES and Development Optimal Level for Social Interaction and Behavior Regulation

The initial key finding of this study was that the socioeconomic context mainly influences the level of development of two of the three early communicative functions studied: SI and BR. Regarding the level of development of social interaction as a communicative function, children in the lowest socioeconomic context (Very low SES) obtained the lowest level of development with respect to their peers in the Low SES and High SES groups, who were at the highest levels of development in these two skills. This suggests that social distances between Very Low and Low SES may be bigger than previously thought. Indeed, for a highly unequal society such as the Colombian, SES scales may not be linear, and the context in which children are raised has an influence on the development of early communicative interactions. In line with the reviewed literature, these results show that early care and socialization practices in low-income countries are shaped by social and economic conditions. This is important because according to sociocultural theories of development, social understanding and social interaction skills are developed from the beginning of life (Werner and Kaplan, 1963; Carpendale and Lewis, 2004). If we consider that in a country like Colombia, children from birth are already in a situation of social inequality, these results could be more understandable, thus, indicating that inequality could possibly translate into unequal social skills.

Results are also aligned with those of Conger and Donnellan (2007) and Leventhal et al. (2015), regarding children’s early social interactions and the detrimental effects that the lack appropriate social and economic conditions can have on them, including diminished stimulation and interaction. Studies have shown that these conditions, typical of severe or persistent impoverishment, in fact, increase stress levels in caregivers, due to the daily struggles these caregivers have to face to secure household resources and to try to cope with life in a deteriorated environment or in dangerous circumstances, but that manifests in children not receiving effective care, thus affecting not only their cognitive but also social development, as explained by Bornstein and Lanslord (2010) and Brito and Noble (2014).

The results further reassert the importance of the initial care of the child in their cognitive and social-communicative development. Nowadays, it is evident that social interaction depends as much on the care the child receives, as on the caregivers’ conditions. However, for this care to be effective in terms of social development it must have the form of what has been called “*nurturing care”*, that includes, for example, the quality, quantity, and adequacy of the child’s nutrition and illness care, attachment and socialization, safety and protection from threats, and, above all, interactions that are emotionally supportive. We hold that this type of care influences interaction and early social communication, which is consistent with the attempt made by Urke et al. (2018) to measure the concept of “*nurturing care*” in Colombia, that resulted in a correlation between mothers’ quality of nurturing care and their maternal resources derived from SES, such as her level of education, household assets, among others.

On the other hand, as very specific cultural practices are related to the age at which children acquire socio-communicative skills in the early years Callaghan et al. (2011), it could be conjectured that the significant differences found in the social interactions between the different socioeconomic groups could be explained by the different explicit socio-communicative practices taught by the adults in each social group. For example, during the administration of the scale to children of the lowest SES level, the Wayuú, we realized that when interactive games were proposed, the majority of these children looked at the adults who accompanied them, seeking their approval to proceed while this was not the case for the other children, who tended to be more independent from their parents while playing with the evaluator. This finding possibly reveals differences in their childcare practices.

Although both Very Low SES (Wayuú) and Low SES children attend childcare facilities, those attended by Wayuú children are directed and designed to guarantee the preservation of their customs and cultural practices that favor traditionalism and hierarchy, where authority has more social weight, even in communication with others. Although we might think that this is a common feature in the development of children in the second year of life, when the results were analyzed by the roles played by children in social interaction, significant differences were found in their response to social interaction (ASI), and this was their lowest indicator. To validate this conjecture, it would be worth assessing the familiarity of children with the proposed games and objects, to eliminate this as a possible factor weighting in the children’s response.

Additionally, regarding the development optimal level for behavior regulation function, differences were also found according to the socioeconomic context. Children in the Very low SES group obtained the lowest level of development with respect to their peers in the Low social and High SES groups. Our findings also show that children in the Low SES group did exhibit an optimal developmental level for the BR communicative function that was even higher than their High SES peers, challenging the idea that the higher the SES, the better the scores. This could be thought of as contrary to the results of Pisani et al. (2018) wherein lower socioeconomic status was correlated with higher levels of child behavior problems, however, their research did not study specifically *Behavior Regulation* as a communicative function under a sociopragmatic interactive perspective, as in our work. It could be argued that our results reveal a gap in BR communicative function research that needs to be addressed.

This gap is a direct consequence of the fact that most of the literature that studies that link behavior regulation and SES mostly focus on emotional regulation, parenting practices and behavior problems, in children older than 3 years old (Flouri et al., 2014; Pisani et al., 2018; Quetsch et al., 2018), and not so much on communicative and language development in interactive settings. Additional research in early childhood development could reveal the actual weight factors such as parental practices in the child’s behavior regulation communicative function.

In summary, from the above it follows that it could be potentially revealing and interesting to further study the regulation of behavior in children before the age of three, as it is becoming more evident that children need to engage with others to learn how to manage their behaviors at these ages, especially since it is only after three years of age that the child expresses more intelligible verbal language. In other words, their regulation will also depend on the adult’s response to their requests. Our study showed that precisely the difference in emotional regulation was more noticeable in the Very Low and Low SES groups and for the sub-items that measure the response to behavior regulation (ABR), with the Very Low SES group evidenced ability to initiate of behavior regulation (IBR) but showing low scores at answering to BR (see results in Table 4). These differences in ABR may be related to the way adults teach how to answer to regulation of behavior and not necessarily to lack on initiation. In fact, in this series of items even the Wayuú children did not have low scores. As behavior regulation skills are learned during the three first years of life and determine the competence level in this function, allowing children to achieve psychosocial adaptation, early behavior regulation teaching programs must include caregivers.

The scale used to assess the communicative development of children in this research is based on Fisher’s neo-Piagetian mode (Fischer, 1980) that includes not only sensorimotor knowledge as described by Piaget (1963), but also knowledge related to language and communication development. Unlike Piaget’s theory, Fisher’s theory attributes a key role to the environment in the appearance and organization of knowledge. The environment would then offer opportunities to exercise a skill or to exercise it in a particular way. In this sense, the interactive skills that are developed in the first two years of life provide a foundation for all subsequent social and communication development. If delays in social-communicative development can be identified early in life and changes can be made in the way the social environment interacts with the child, timely interventions can effectively facilitate social development (Mundy et al., 2003).

### SES and Development Optimal Level for Joint Attention

A second key finding in this research was that the socioeconomic context did not have a significant effect on the development of Joint Attention as we had hypothesized in this study. Based on the cultural studies of Liszkowski et al. (2012) that had supported the idea of universality of joint attention (measured through the gestures of pointing in 7 cultures), we also proposed in this study, the universality of joint attention based in the SES. We assumed that the SES would not exert any influence on the level of development of joint attention, and in fact, we did not find significant differences between the groups.

We consider that this result was key in our study for two fundamental reasons. First, this communicative function was the only one that did not show significant differences between the groups; let us remember that significant differences between groups (the very low and high for the SI and between very Low and Low for the regulation of behavior) were notable for the social interaction and the regulation of behavior. Second, this communicative function has been one of the most studied and has been considered key for the development of language, pointing out its importance through the hypothesis of language continuity.

This idea of linguistic continuity of language has been questioned by some authors who argue that the assumption that joint attention is a necessary and sufficient precursor to vocabulary learning is not universally supported (Akhtar and Gernsbacher, 2007). Although this study presents interesting criticisms, especially for the measurement of joint attention (it is only evaluated in the visual modality), it is clarified that the idea in its criticisms is not to challenge the correlation between joint attention and the development of vocabulary, but to critically examine the generality of that correlation and to confront the assumption that the relationship between joint attention and vocabulary development is causal. Although the objective of our study was not to search for causal correlations, it is based on the studies of Bruner (1983) and the usage-based theory of language acquisition, proposed by Tomasello (2003), who defends the idea of continuity of language and the use of a functional or pragmatic approach for understanding communicative development. However, we support Akhtar’s idea that future joint attention studies should not only consider visual indices for their evaluation.

On the other hand, the idea of universality can also be supported from an evolutionary perspective. Tomasello et al. (2005) showed that although some non-human primates understand more about intentional action and perceptions than previously believed (and this is also true, to some extent, in children with autism, Thommen et al., 2016), only human children engage socially and culturally with others. In other words, these studies show that understanding the intentional actions and perceptions of others is not enough by itself to produce human-like social and cultural activities, it requires shared intentionality. The hypothesis defended by these researchers is that only human beings are biologically adapted to participate in collaborative activities that involve shared objectives and socially coordinated action plans (joint intentions). In this sense, the usage-based theory of language acquisition is also compatible with interactionist perspectives on the development of language where individuals are actors of their intentions, these intentions being visible and interpretable from an early age (Guellai and Streri, 2011).

Studies relating socioeconomic context to language development have almost always found differences in context and a negative effect especially in children living in low-income areas (Arriaga et al., 1998; Hoff, 2003). De los Reyes et al. (2016) looked at 629 children (0–5 years) living in low-income rural areas in northern Colombia and suggested that impoverished social contexts do offer opportunities that favor the development of the social domain but restrict the development of the cognitive domain. Our study is consistent with the results of De los Reyes et al. (2016) regarding the effect of the socioeconomic context on the communicative functions of social interaction and behavior regulation; however, it differs in that there is no effect of this context on the specific level of joint attention.

Our results seem to point more toward studies that admit universal mechanisms in the origin of joint attention, in children of 2 years. However, we studied the socioeconomic context and not explicitly culture and although these two concepts are related, when studying language development, care must be considered as suggested by Sabatier (2014) in his theoretical review on the contribution of cultural psychology to developmental modeling. We believe that our results are very valuable and consistent with some studies, but we also believe that future research should consider more sociocultural variables.

Finally, our results are based on a relatively small sample and as such there is ample scope for future research to consider larger samples and/or longitudinal methods. Such research could help determine whether joint attention is universal or if it is universal practices in child upbringing that may be responsible for the equal development of joint attention around the world.

### Low SES and Development of Communicative Functions

Finally, we want to highlight one last result. We found that the low SES group always obtained the expected level of development in all communicative functions, and even for behavior regulation they obtained the highest optimal level with respect to their peers in the high SES group. This suggests that the minimum required guaranteed resources and interactions necessary for an individual to develop an average skill performance may not be that high after all, and also challenges the idea that the highest SES group always obtained the highest scores. We consider that this result contradicts the idea that unequivocally SES level and scores are directly and almost causally related, which may be wrongly inferred from studies such as those presented in the theoretical background review of this research. Although evidence for a high correlation between SES and language development is undeniable, perhaps future studies should further analyze these kinds of results in the light of other variables that could factor in the overall performance.

In our study, children with low SES attended governmental non-profit early childcare facilities named “ICBF Child Development Centers (CDI).” These centers, as explained, provide low-income children under 5 years of age with initial education, care, and nutrition, within the framework of Comprehensive and Differential Care. These centers implement pedagogical, qualified care and nutrition practices, as well as take steps to promote health, protection, and participation rights, which allow for the integral development of children. Results for this group allow us to deduce that these experiences are positive in children and could have influenced their performance in the evaluation. In addition, recent studies developed in Colombia to evaluate these childcare programs have shown both the effectiveness of these centers, as well as the positive influence on parents who educate and regulate the behavior of their children in proper ways (Urke et al., 2018; Lopez-Avila, 2019). In line with these ideas, the fact that the High SES children obtained a lower score in the level of development of behavior regulation with respect to the Low SES group could also be explained by the fact that these children are cared for by babysitters and most are homeschooled with parents who work and are not around most of the time. When children attend these childcare centers, they share with other children and may have more opportunities to learn to be communicatively competent.

We found that the level of development of social interaction and behavior regulation communication skills is always lower in the most socially vulnerable social context (Very Low SES). This last result does not seem to bring anything new, as previously indicated in most studies, being born, and growing up in environments of poverty and extreme poverty constitutes a very high risk for the development of the child in all their dimensions, especially in terms of cognitive and language development. However, we believe that at least for the evaluated sample, the poorest (that is, the group of children belonging to the Very Low SES, the Wayuú) did not differ from the others in terms of joint attention. Although it is true that group selection and assessment has merit on itself focusing exclusively on SES differences, it is also true that by these children belonging to an indigenous community with extreme poverty levels and a culture that is different from non-members of their tribe, any supporting evidence for the inexistence of significant differences in joint attention for this group would be indicative of the assertiveness of the hypothesis that JA development is in fact universal, as Wayuú differ vastly from their peers not only terms of SES and but also in their cultural background.

Only limited literature exists in terms of evaluating this hypothesis, and what is available tends to focus on how the environment impacts on language development, not on early communicative functions. If delays in social-communicative development can be identified early in life and changes can be made in the way the social environment interacts with the child, an intervention could effectively facilitate social development (Mundy et al., 2003). Such interventions need to be properly strategized, and to do so, it is necessary to understand which variables can be subjected to changes that will positively address conditions that translate into an overall improvement in children’s prelinguistic communicative functions scaffold.

### One Final Thought

In this work, the communicative functions of 24-month-old children were assessed through a cross-sectional study. However, results indicate the need to undertake further research involving a variety of factors, such as parents’ educational level, parental occupation, time spent by parents/caregivers on interacting with children, parental and caregiving practices, communicative context of children, location, and even ethnic differences, to assess communication skills in children through longitudinal studies. An important step forward from our findings, that address the cultural aspect by pondering the impact of the socioeconomic context that differentiates these children, would be an in-depth study into the influence of sociocultural experiences on the development of communication and language.

As known, in Latin American countries SES marks the way in which children develop culturally. However, we believe that our study can contribute to developmental psychology in terms of reflecting on both the universalities as well as the particularities of children’s development around the world. As Koller and Araujo De Morais (2018) argue, referring to Latin America, “the emphasis on the strategies used to overcome adversity and face daily challenges would constitute a new paradigm in the study of human development” (p. 87). In this way, we can incorporate more positive and less dire perspectives into studies of children’s communication and language development.

## Data Availability Statement

The raw data supporting the conclusions of this article will be made available by the authors, without undue reservation.

## Ethics Statement

The studies involving human participants were reviewed and approved by the Ethics committee of the Universidad del Norte in Colombia (registration number 185-28-02-2019). Written informed consent to participate in this study was provided by the participants’ legal guardian/next of kin.

## Author Contributions

MM and MG made a key contribution to design the study, analyze, interpret, and discuss the data. MM wrote the manuscript. MG and ET made a key contribution to the design and revision of the manuscript. EM made a key contribution in the collection and coding of the data. All authors contributed to the article and approved the submitted version.

## Conflict of Interest

The authors declare that the research was conducted in the absence of any commercial or financial relationships that could be construed as a potential conflict of interest.
